# Evaluation of color matching and microhardness of two different universal-shade resin composites: an in-vitro study

**DOI:** 10.1186/s12903-025-06424-1

**Published:** 2025-07-03

**Authors:** Khaled Mahmoud Hijazi, Ahmed Fawzy Abo Elezz, Amr Faisal Ghonaim

**Affiliations:** Suez Canal University, Ismaileya, Egypt

**Keywords:** Color, Composite, Microhardness, Shade, Staining, Thermocycling

## Abstract

**Background:**

Dentists have started to use universal shade resin composites to simplify shade selection. This in vitro study assessed the color matching of two distinct universal shade resin composites (OMNICHROMA^®^ and ONEshade^™^) following immersion in staining solution and their microhardness during thermocycling.

**Methods:**

For color matching evaluation, forty maxillary sound human premolars were collected. Only A3 shaded teeth were chosen after VITA Easyshade V recorded the color coordinates of the teeth via the CIELab color system before interventions. After that, the teeth were randomly allocated into two groups according to the tested materials, with twenty teeth included in each group (*n* = 20). The teeth were then prepared with class V cavity preparations, the group (M1) teeth were restored with Omnichroma, and the group (M2) teeth were restored with Oneshade. All teeth were evaluated after 24 h of restoration (T1), after 3 months of immersion in tea solution (T2) and after 6 months of immersion in tea solution (T3). To evaluate color matching, both instrumental and visual color changes were recorded. For microhardness evaluation, forty cylindrical discs were made (20 discs for each composite group M1 and M2). Vickers microhardness measurements were performed via a microhardness testing machine. The samples were measured at baseline (C1), after 2500 thermocycles (C2) and after 5000 thermocycles (C3).

**Results:**

In accordance with the color matching results, both tested composite materials showed significant color mismatching after immersion in tea solution. In accordance with the surface microhardness evaluation, both tested resin composite materials showed a significant decrease in the Vickers microhardness after thermocycling.

**Conclusions:**

Following immersion in the staining solution, both evaluated resin composite materials displayed unsatisfactory color matching values in class V restorations. Accelerated aging by thermocycling had an obvious negative effect on the microhardness of the universal shade resin materials.

## Background

Dental resin composites are considered the most popular direct tooth-colored restorations used for restoring cervical lesions as class V cavities [1]. Resin composite restorations should simulate normal tooth structures in terms of color parameters and optical properties [2]. The polychromatic nature of sound teeth makes composite shade selection and matching more difficult [3]. 

Traditional resin composites include a range of shades that require careful matching to the patient’s natural teeth, which can be time-consuming and difficult. Universal shade composites were developed to overcome this challenge, as, according to the manufacturers, these composites come in a single shade that matches all the classical shades from A1 to D4 [4]. The development of these materials has had a significant impact on clinical practice, particularly in terms of simplifying procedures and improving outcomes. Universal shade composites minimize the need for clinicians to select a specific shade for each part of a tooth, reducing the time spent on shade selection and increasing clinical efficiency. Patients expect restorations that look natural and indistinguishable from their original teeth. Universal shades meet this demand by blending seamlessly with natural tooth colors, making them excellent choices for aesthetic restorations. Universal shade resin composites can be used for a wide range of restorative procedures, from Class I to Class V cavities, veneers, and direct restorations in both the anterior and posterior regions. This versatility makes them a go-to option for many clinicians, reducing the need for a broad range of different composite materials [5]. 

Various beverages, including water, wine, tea, coffee, and soft drinks, have been used to assess the staining effect of solutions on the color matching of resin composites. Depending on the composition and test solutions used, these beverages exhibit a variety of discoloration [6]. To measure color matching accurately, both instrumental and visual methods should be used whenever possible because they supplement each other and can lead to prospective esthetic results [4]. 

The optical properties and color matching technology of these recently introduced composite materials have received the most attention from the scientific community. Even if manufacturers and independent researchers have provided some information regarding the mechanical durability of universal shade composites, any restorative specialist is still highly interested in this topic. In particular, the mechanical properties following the accelerated aging of these composite materials should be investigated [7]. 

According to what was mentioned above, it was interesting to assess how the staining challenge affects the capability for color matching, in addition to investigating how thermocycling affects the surface microhardness of these new, aesthetically appealing composite materials. The null hypotheses were as follows: first both tested resin composites had acceptable color matching after immersion in tea solution, and second the tested resin composites showed no difference in microhardness after thermocycling.

## Materials and methods

### Materials

- Dental composite resin materials: Omnichroma/Oneshade (Table 1).
- The beverage utilized was black tea (Lipton Yellow Label, Unilever, Egypt).

**Table 1:** Materials used in the study

Materials	Description	Composition	Manufacturer	Batch number
Omnichroma	Universal shade resin composite	Filler System: 79% by weight (68% by volume) of spherical silicazirconia filler (average particle size: 0.3 μm, range: 0.2 to 0.6 μm) and composite filler. Resin System: UDMA, TEGDMA, Dibutyl hydroxyl toluene, Mequinol and Ultraviolet absorber.	Tokuyama Dental, Tokyo, Japan	023E61
Oneshade	Universal shade resin composite	Filler System: 75% by weight (53% by volume) of silicon dioxide, Glass powder and inorganic filler (particle size range: 0.005 to 3.0 μm). Resin System: diurethane dimethacrylate, Bis-GMA, tetra-methylene dimethacrylate.	Olident, Krakow, Poland	2,021,002,502

UDMA, urethane dimethacrylate; TEGDMA, triethylene glycol dimethacrylate; Bis-GMA, Bisphenol A-glycidyl methacrylate

### Methods

#### Study design

For color matching evaluation, forty A3 extracted maxillary sound premolars were randomly allocated into two groups on the basis of the materials that were examined. (M). In the M1 group, the teeth were restored with Omnichroma, and in the M2 group, the teeth were restored with the Oneshade resin composite. Then, the tooth samples were immersed in the tea solution. The instrumental and visual color changes were evaluated at four different time points (T), after 24 h of restoration (T1), after 3 months of immersion in tea solution (T2) and after 6 months of immersion (T3). For microhardness evaluation, forty cylindrical discs were made (20 discs for each composite group, M1 and M2). The samples were subsequently subjected to thermocycling. Vickers microhardness measurements were performed via a microhardness testing machine. The samples were measured at baseline (C1), after 2500 thermocycles (C2) and after 5000 thermocycles (C3). The research proposal was reviewed and waived by the Ethics Committee of the Faculty of Dentistry, Suez Canal University, Egypt (#365/2021).

#### Sample size calculation

Two way analysis of variance (ANOVA) was proposed. A total calculated sample size of 80 samples was sufficient to detect the effect size of 0.55 according to Cohen, a power (1-β = 0.80) of 80% at a significance probability level of *p* < 0.05 partial eta squared of 0.21. According to sample size calculations each subgroup would be represented by 20 samples with a total sample size of 80 samples. The sample size was calculated according to G*Power software version 3.1.9.3.

### Color matching evaluation

#### Tooth selection

This study was carried out on 40 sound, freshly extracted human maxillary premolars obtained from patients undergoing extraction due to periodontal diseases. Immediately following the extraction procedure, to remove blood and mucous, the teeth were properly washed under flow water and scaled to remove calculus and any remaining periodontal ligaments. Then, the teeth were polished with fine pumice and soft rubber cups [8]. A magnifying lens was used to check for cracks in the teeth. Every tooth that showed any indications of fluorosis, caries, microcracks, restorations, or other cervical abnormalities was replaced [9]. Before use, the forty selected teeth were kept at room temperature in distilled water containing a 0.5% chloramine-T antiseptic solution [10]. 

#### Preoperative procedures

Prior to starting cavity preparation, the color of each tooth was measured via a spectrophotometer (VITA Easyshade V^®^, VITA Zahnfabrik, Germany). All teeth in this study had the same shade (A3), and other shades were replaced. On the basis of the materials utilized in the study, teeth were randomly allocated into two groups (M1 and M2).

#### Class V preparation and restoration

On the buccal surface of every tooth, a standardized class V cavity preparation measuring 4 mm in width, 2 mm in length, and 1.5 mm in depth was established. 0.5 mm coronal to the cementoenamel junction [11]. 

Cavities were prepared via the use of a fissure carbide bur (No. 56, Komet Dental, Lemgo, Germany), which was mounted on a high-speed contra angle hand piece with a copious water spray. A 45° short bevel was prepared on the cavity’s occlusal wall using a diamond fissure bur (SF 110 − 014, Mani, Tokyo, Japan). The bur was replaced by a new one after every 5 cavities in order to ensure high cutting efficiency. The final dimensions were checked via a periodontal probe (Williams 568/1, Medesy, Maniago, Italy) [12]. 

Cavities were etched with 37% phosphoric acid etchant gel (super etch, SDI Limited, Victoria, Australia Australia) for 20 s and then rinsed for 30 s. After air drying, a double layer of the adhesive bonding agent (All-Bond Universal, BISCO Inc., Schaumburg, IL, USA) was applied in accordance with the recommendations of the manufacturer to the etched cavity with a rubbing motion for 20 s, then lightly air thinned with air flow for 2–5 s and light cured for 10 s with a light curing unit (Elipar B10, 3 M ESPE, St. Paul, MN, USA) working in standard mode at 1200 mW/cm^2^ light intensity [13]. 

The tested resin composite materials were bulk packed via a Teflon-coated plastic filling instrument, and the final layer of the restoration was covered with a transparent polyester strip (TOR VM, Moscow, Russia) and then light-cured for 20 s to reduce the oxygen inhibition layer and achieve the smoothest surface possible. The curing distance was standardized by applying the tip of the light curing device on the tooth’s buccal surface. A radiometer (Demetron LED Radiometer, Kerr Corp., CA, USA) was used to measure the light curing irradiance every 5 uses [10]. 

All the samples were finished and polished via gentle unidirectional strokes with aluminum oxide discs (Sof-lex, 3 M ESPE, St. Paul, MN, USA) for 15 s in dry medium, followed by 6 s of rinsing and drying with an air/water syringe. The samples were kept at room temperature in tightly sealed vials filled with distilled water for 24 h [12]. 

#### Staining protocol

The black tea (Lipton yellow label, Unilever, Cairo, Egypt) used in this investigation was made by soaking 8 g of loose tea in one liter of boiling water for five minutes. Before the prepared tea was poured into containers, any contaminants were removed via filter paper. Each tooth sample was immersed individually in containers filled with 20 mL of staining solution. To prevent any bacterial or fungal contaminants, the solution was freshly prepared every day, and the samples were submerged in the tea solution for 15 min once daily. After the immersion regimen, the samples were washed and kept in distilled water at room temperature [14]. 

### Color parameter testing

**Fig. 1:**
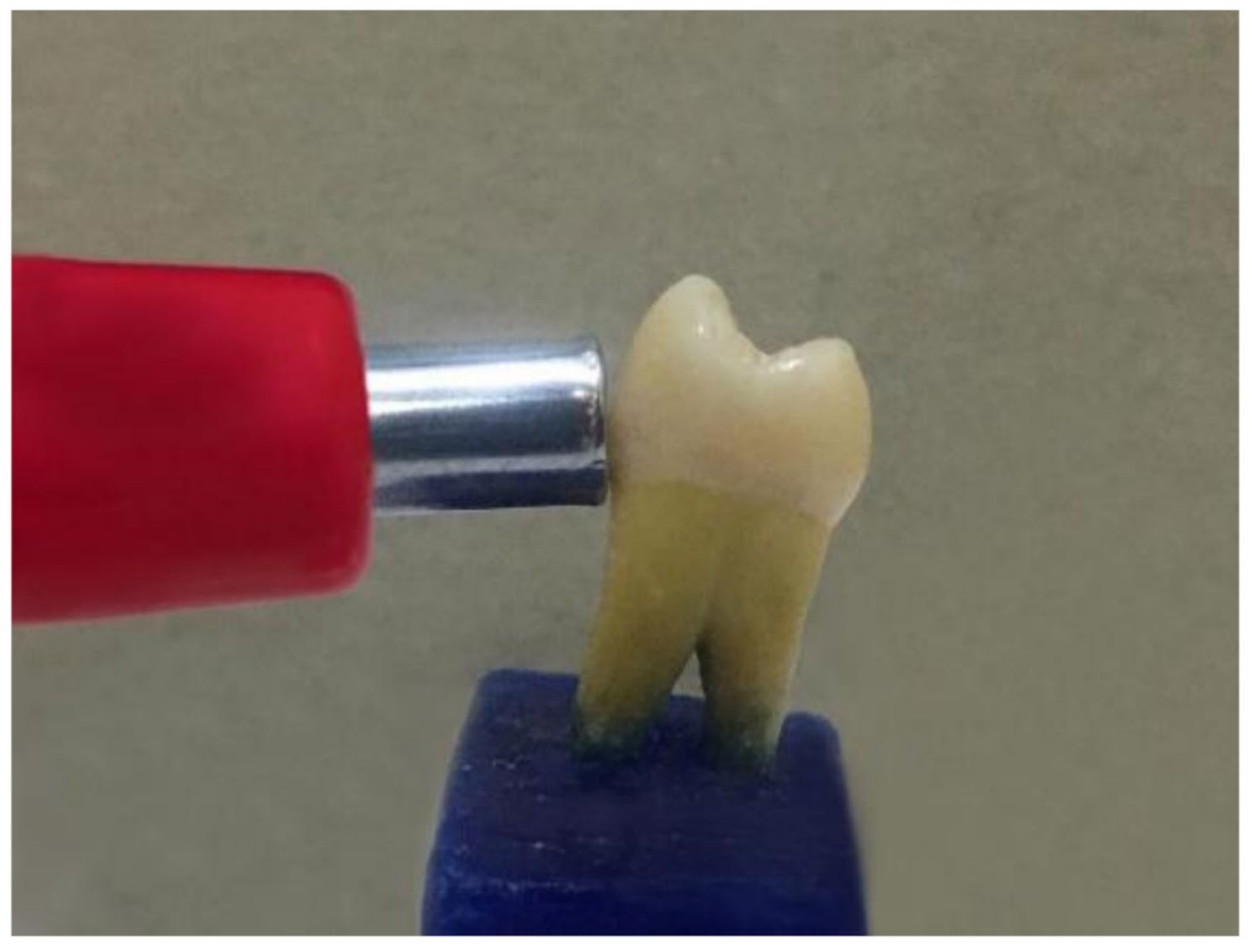
Color measurements were taken using a spectrophotometer (VITA Easyshade^®^ V Vita, Zahnfabrik, Bad Sackingen, Germany)

Both objective and subjective evaluation methods were used to evaluate the color matching of the tested materials. Objective instrumental evaluation was performed with a VITA Easyshade V spectrophotometer based on the CIELab color system (Fig. 1), whereas subjective visual evaluation was performed via visual analysis [15]. 

### Instrumental color evaluation

Before the measurements were taken, the teeth were stabilized in dental wax molds to increase the ease of handling. Then, the buccal surface of each tooth was rinsed with water and wiped off with gauze to prevent any sliding. The first step in Vita Easyshade measurement procedures was a calibration procedure carried out in accordance with the manufacturer’s guidelines. The device was set at “Tooth area shade determination” mode. This measurement mode enables the determination of the shade in the cervical, central and incisal areas of the buccal surface of a tooth individually, with the measuring tip placed 90° relative to the tooth and laying flushed on each area [12]. 

Three consecutive color measurements were made to obtain an accurate measurement. At each time, the obtained values of the cervical class V restoration and tooth surface at the middle third 1 mm away from the restoration margin were transformed into the compatible L, a, and b values that the manufacturer had provided, and the average of the three readings was calculated [16]. 

The color matching was evaluated by calculating the color difference (ΔE), which represented the total or the overall color difference between the restoration (cervical third) and the tooth itself (middle third). The following formula was used to calculate the ΔE values according to the CIELAB color system: [17–20]

$$ {\rm{\Delta E}}\,{\rm{ = [(\Delta L*}}{{\rm{)}}^{\rm{2}}}{\rm{ + }}\,{{\rm{(\Delta a*)}}^{\rm{2}}}{\rm{ + }}\,{{\rm{(\Delta b*)}}^{\rm{2}}}{{\rm{]}}^{{\rm{1/2}}}} $$

where L* represents the color lightness parameter, a* represents the color redness‒greenness parameter, and b* represents the yellowness‒blueness parameter of the color. ΔE ˃3.7 is regarded as clinically undesirable, whereas ΔE ≤ 3.7 is regarded as an acceptable clinical color change [21]. 

### Visual color evaluation

Under a D65 light source and a distance of 25 cm from the samples, visual color matching assessment was performed by twenty dental teaching assistants with normal color vision as determined by the Ishihara color blindness test. To assess the color matching between the tested composite materials and the tooth shade, the visual scoring (VS) values were numerically represented as follows [19]: (0) perfect match, (1) very good match, (2) borderline match, (3) clear mismatch, and (4) severe mismatch.

### Microhardness evaluation

#### Specimen preparation

**Fig. 2:**
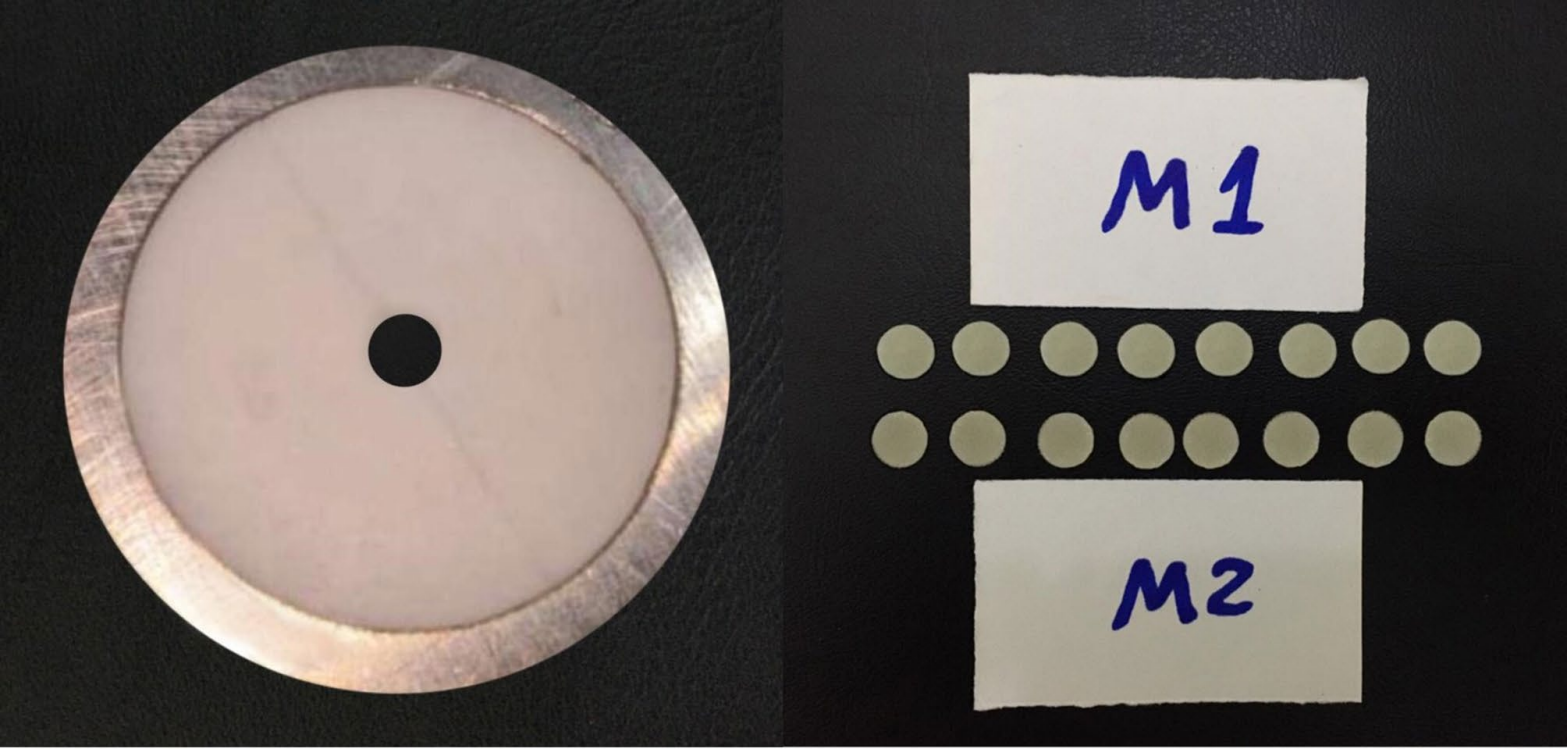
Preparation of resin composite discs using Teflon split mold

Twenty disc samples (6 mm in diameter × 2 mm in height) from each group were prepared via a cylindrical Teflon split mold (Fig. 2). The Teflon mold was positioned on a transparent polyester strip over a glass slab (1 mm thick) and filled in one layer with the resin composite that was tested. To minimize the oxygen-inhibiting layer and enhance the surface quality of the composite sample, a glass slab and another transparent polyester strip were placed over the surface of the mold. To ensure proper packing, obtain parallel surfaces and allow extra material to drip out, the mold was pushed with a finger and then loaded with 300 g weight for 30 s [22]. The samples were then light cured for 20 s on both the top and bottom surfaces to ensure optimal curing and enhance the material properties. To standardize the curing distance, the curing unit’s light tep was flushed on a glass slide [15]. 

A digital caliper (ABSOLUTE Super Caliper Series 500, Mitutoyo, Tokyo, Japan) was subsequently used to evaluate the dimensions of the samples. Finishing and polishing of the prepared samples were performed with aluminum oxide discs as in the color matching test. For rehydration and complete polymerization, the samples were subsequently submerged immediately in distilled water at room temperature for twenty-four hours before the baseline microhardness evaluation was performed [23]. 

#### Thermocycling protocol

After the baseline microhardness measurements, the samples were subjected to the thermocycling process in a Thermocycler (Thermocycler THE 1100, SD Mechatronik, Feldkirchen-Westerham, Germany), which uses cyclical temperature alternations between 5 °C and 55 °C distilled water baths with a 30-second dwell time per bath and a 10 s transfer time to replicate artificial aging. Microhardness was measured after 2500 cycles and after 5000 cycles, since according to studies, In-vitro thermocycling to 2500 cycles and 5000 cycles equals 3 months and 6 months of intraoral service, respectively [24]. 

#### Microhardness testing

The Vickers microhardness of all the samples was measured via a digital display Vickers microhardness testing machine (Wilson Tukon 1102, BUEHLER, Leinfelden-Echterdingen, Germany) equipped with a 50X objective lens and a Vickers diamond indenter. Following ten seconds of applying a 100-gram force to each sample surface, three evenly spaced indentations that were no closer than 0.5 mm apart were formed on each sample surface. The following formula was used to determine the microhardness: VHN = 1.854 P/d2, where d is the diagonal length in mm, P is the load in kgf, and VHN is the Vickers hardness in kgf/mm2. Each group was evaluated 3 times according to the number of thermal cycles (C): before thermocycling (C1), after 2500 cycles (C2), and after 5000 cycles (C3) [25]. 

### Statistical analysis

All of the collected data were noted, tallied, and examined for normality. The results of the Vickers microhardness, ΔE, and visual color matching were examined via one-way analysis of variance (ANOVA) and Duncan’s multiple range tests (DMRTs). The threshold for statistical significance was established at *p* < 0.05. IBM^®^ SPSS^®^ for Windows version 22.0 software (IBM Corp., Armonk, NY, USA) was used to statistically analyze the data.

## Results

### Color measurement results

Table 2 shows the means and standard deviations of both the instrumental color matching (color changes ΔE) and visual scoring (VS) values of different materials (M1 and M2) at different time points (T1, T2 and T3). One-way repeated-measures ANOVA revealed a highly significant difference (*p* < 0.001) between time points within groups M1 and M2. ANOVA was followed by Duncan’s post hoc test. Furthermore, Duncan’s multiple range test (DMRT) at the 0.05 level revealed that the means followed by distinct letters were significantly different.

**Table 2:** The mean and standard deviation values of ∆E and VS for the tested resin composites

Variables	∆E				VS			
	M1		M2		M1		M2	
	Mean	SD	Mean	SD	Mean	SD	Mean	SD
T1	4.31^c^	0.44	4.58^c^	0.58	1.91^c^	0.04	1.93^c^	0.04
T2	5.07^c^	0.26	5.01^c^	0.55	2.72^b^	0.04	2.74^b^	0.04
T3	7.17^b^	0.5	7.23^b^	0.45	3.22^a^	0.09	3.41^a^	0.09
*P* value	< 0.001***		< 0.001***		< 0.001***		< 0.001***	

*ΔE, color difference; VS, visual score; M1, Omnichroma; M2, Oneshade; SD, standard deviation

### Microhardness results

The mean and standard deviation values of the Vickers microhardness for different materials (M1 and M2) and different thermocycling intervals (C1, C2 and C3) are shown in Table 3. One-way repeated-measures ANOVA revealed a highly significant difference (*p* < 0.001) between the thermocycling intervals of groups M1 and M2. ANOVA was followed by Duncan’s post hoc test. Furthermore, Duncan’s multiple range test (DMRT) at the 0.05 level revealed that the means followed by distinct letters were significantly different.

**Table 3:** The mean and standard deviation values of Vickers microhardness for the tested resin composites

Variables	M1		M2	
	Mean	SD	Mean	SD
C1	40.74^a^	2.38	40.09^a^	1.72
C2	37.90^b^	2.20	38.28^b^	2.09
C3	36.57^c^	1.98	37.04^c^	1.09
*P* value	< 0.001***		< 0.001***	

* M1, Omnichroma; M2, Oneshade; SD, standard deviation

## Discussion

The widespread use of various resin composite color shades for anterior and posterior restorations has garnered significant interest because of their ability to mimic natural tooth aesthetics. However, the selection and application of these materials often lead to extended chair time, presenting challenges for dentists [26]. These extended procedures not only require a high level of skill but can also cause discomfort for patients and increase the overall treatment time. New single shade resin composites have been recently released by several manufacturers since they reduce the chair time and shade selection time [12]. By producing a red-to-yellow structural color that is comparable to the color of natural teeth, these new composite materials accomplish wide color matching capability. Therefore, assessing the capacity of these contemporary aesthetic composite materials for color matching was deemed fascinating [27, 28]. 

Two different universal shade resin composites were used in the current study depending on the different compositions: the type and size of the fillers and the distributed resin matrix. A commercially available nanofiller resin composite (Omnichroma) was compared with a microhybrid resin composite (Oneshade) [1]. 

Teeth with A3 shade were selected for this study since, when considering the overall distribution of tooth shades in the human population, A3 tends to fall within the middle of the typical range of tooth colors, and the A3 shade provides moderate contrast to various staining agents (e.g., coffee, tea, and tobacco) without being too extreme in either direction. This makes it easier to observe the effects of these agents over time, as both lighter and darker shades may not show gradual changes in the same way [18, 29]. 

This study followed the methodology used in previous studies [10, 11, 15, 30, 31]. All possible efforts were made to guarantee the standardization of the methodology and that the same operator carried out all of the procedures [30]. Class V cavities were selected in this study because (a) the margins of class V restorations are located in enamel as well as in dentin and (b) the restoration is influenced by the color of the dentin because this region has a thin enamel thickness [11, 32]. 

Moreover, this study highlighted the problem of resin composite color mismatching when exposed to commonly consumed beverages. The staining solution used in this study was black tea. This solution was chosen as the most popular daily beverage with the potential to stain and covers a wide range of staining shades due to the involvement of tannic acid and stains in its ingredients [16]. 

Color matching evaluation was performed visually and instrumentally, and spectrophotometry may be more reliable and provide an objective method for in vitro studies. VITA Easyshade V is recommended for in vitro investigations as the most reliable, sensitive and accurate device with up to 90% accuracy [17, 29, 33–35]. 

The color system CIELab, which was employed in this study, is highly suggested for dental purposes. Three spatial color coordinates—L*, a*, and b*—are used by the system to determine color on the basis of human perception. ΔE represents the total color difference, and a ΔE value of 3.7 is considered an acceptable threshold on the basis of data on acceptability and perceptibility thresholds found in the dental literature. Numerous studies have utilized this value as a standard reference [8]. 

Although visual analysis records are influenced by the perceptions and choices of participants, it remains the most common qualitative method used to evaluate the color matching and esthetic improvement of dental restorations in most trials [4, 10, 15, 31]. 

In the present study, the two tested composite materials showed statistically significant color mismatching to the tooth structure visually and by instrumental evaluation (∆E > 3.7) after 3 and 6 months of immersion in tea solution. Our results were in accordance with those of ***Ersöz*** et al.., who reported that a single shade resin composite had greater discoloration potential than multiple shade resin composites did after 30 days of immersion in tea [36]. Additionally, our results were in accordance with those of ***Fazlioğlu*** et al., who reported that the ΔE values of universal shade composites were above the clinically acceptable value after immersion in tea for 24 h every day for 6 days [37]. 

In contrast, ***Kalander*** et al.. reported that disk-shaped samples made from Omnichroma resin composite material exhibited less color change than the other tested composite materials did after immersion in beverages with different stains, such as tea, coffee, orange juice, and cola, for 28 days [20]. These contradictory results might be due to the tested materials being evaluated as disk-shaped samples rather than as restorations in extracted human teeth.

Universal-shade composites are engineered to reduce the need for shade selection by utilizing optical blending mechanisms. OMNICHROMA employs structural color technology, wherein its uniform supra-nano spherical fillers (~ 260 nm SiO₂-ZrO₂) reflect specific wavelengths (mainly in the red-yellow region) to match surrounding tooth structure without the use of added pigments. Conversely, ONEshade utilizes a pigment-based chameleon effect, relying on the partial transmission and diffusion of surrounding shades to achieve visual integration [38]. 

Despite their different blending mechanisms, both materials showed clinically significant color mismatch (ΔE > 3.7) following tea immersion. In OMNICHROMA, the color stability is dependent on the integrity of light scattering mechanisms through the filler network. Tea contains chromogenic compounds such as tannins and polyphenols, which likely penetrated the hydrophilic resin matrix (comprising UDMA and TEGDMA), thereby disrupting the filler-matrix interface and altering light reflection. Such interference in light scattering likely impaired the structural color effect, reducing the composite’s ability to blend with adjacent tooth shades [1, 39, 40]. 

In ONEshade, the color change is attributed to extrinsic stain adsorption and absorption by the resin matrix and incorporated pigments. The presence of Bis-GMA and UDMA in the matrix composition contributes to water sorption and facilitates pigment penetration, resulting in a shift in optical properties. Furthermore, surface irregularities or insufficient polishing could enhance the retention of staining agents, exacerbating discoloration in both materials. These findings align with prior studies indicating that both structural and pigment-based universal composites are susceptible to discoloration when exposed to dietary chromogens, especially under prolonged immersion or poor oral hygiene conditions [36, 38, 41]. 

Numerous mechanical characteristics should be considered when evaluating recently released dental resin composite restorative materials. Among these properties is the surface microhardness, which is closely related to the wear resistance and consequently the aesthetic properties of the material, as well as how its mechanical and aesthetic characteristics are affected by temperature changes. The literature contains limited and inadequate details about the clinical effectiveness and mechanical characteristics of these materials [42]. To fill this research gap, the current study aimed to assess the surface microhardness of two different universal shade resin composite materials before and after thermocycling.

The thermal range of thermocycling was 5 °C and 55 °C, as the temperature in the oral cavity varies. The mean mouth temperature changes by approximately 36 °C, with the lowest estimated temperature being approximately 5 °C and the highest temperature being over 50 °C. Additionally, the dynamic temperature fluctuations in the thermocycling process are better able to simulate clinical situations and could be highly valuable in evaluating the clinical efficacy of dental resin composite materials [43]. 

Compared with that measured before thermocycling, the surface microhardness in this study showed a significant decrease in the VHN of both tested resin composite materials under the influence of thermocycling for 2500 cycles and 5000 cycles.

Our results agreed with those of ***El-Refai***, who reported that, with respect to the surface microhardness, a significant decrease in the Vickers hardness of an Omnichroma composite was observed after thermocycling compared with that measured at 24 h [42]. Additionally, our results correspond with those of ***Shalaby*** et al., who reported that Omnichroma exhibited a marked decrease in microhardness after thermocycling in distilled water [43]. However, ***Atali*** et al. reported that the microhardness of single shade resin composite materials significantly increased after 15 days of water aging at 37 °C (± 1 °C) [44]. This conflict may be a result of differences in methodology, as they performed static water aging without any changes in temperature, whereas in our study, we used dynamic thermocycling aging at 5/55°C to mimic clinical conditions.

Surface microhardness reflects the material’s resistance to indentation and is an indirect indicator of wear resistance and long-term mechanical performance. Both OMNICHROMA and ONEshade exhibited a significant reduction in Vickers microhardness after thermocycling, which simulates repetitive thermal stress associated with fluctuating intraoral temperatures (e.g., hot and cold beverages) [45]. 

Thermocycling induces hydrolytic degradation and thermal fatigue, particularly at the filler-matrix interface. In OMNICHROMA, the relatively high content of TEGDMA increases the material’s water sorption and plasticization potential, leading to matrix softening [7, 46]. 

Additionally, thermal expansion mismatch between the inorganic fillers and the organic matrix causes stress accumulation, resulting in microcrack formation or filler debonding. These changes compromise the composite’s structural integrity and reduce its surface hardness [47]. 

Similarly, in ONEshade, although its matrix is less hydrophilic due to the presence of Bis-GMA, it still undergoes hydrolytic stress during thermal cycling. Water uptake, filler leaching, and breakdown of the silane coupling agents lead to reduced filler-matrix adhesion and surface degradation. This contributes to the observed decrease in hardness. These results are consistent with previous literature, where resin composites exposed to thermocycling demonstrate reduced hardness and altered surface characteristics [43, 48]. 

Therefore, the two null hypotheses of the study are as follows: (1) both tested composites accept color matching after immersion in tea solution, and (2) the tested resin composites show no difference in microhardness after thermocycling and are rejected.

### Clinical implications

The observed degradation in esthetic and mechanical properties of both composites highlights important clinical considerations. While universal-shade composites offer convenience and simplified shade selection, their long-term color stability and mechanical performance are challenged under conditions simulating oral environment stressors. Patient dietary habits (e.g., consumption of tea, coffee, or red wine) and oral hygiene practices significantly influence the esthetic longevity of such materials. Moreover, surface polishing protocols and restoration placement in high-stress areas may influence the rate of degradation [38]. 

To clinically optimize the performance of universal shade resin composites, maintaining their color match and microhardness over time, proper material selection, effective finishing and polishing, and patient education on the potential for staining after composite restorations; avoiding or minimizing the consumption of stains such as tea, coffee, and wine; and monitoring restorations over time to assess the color and integrity of the restorations and periodic polishing to maintain smooth surfaces and prevent the buildup of surface stains are key factors in achieving successful long-term restorative outcomes [49]. 

### Limitations and future directions

This in vitro study provides valuable insights but is inherently limited by the absence of dynamic oral conditions such as saliva, enzymatic activity, and mechanical wear from mastication. Future studies should incorporate multi-factorial aging models, including brushing abrasion and pH cycling, to better simulate the oral environment. Additionally, evaluating other universal-shade materials could provide comparative data for more informed material selection in clinical practice.

## Conclusions

With respect to the limitations of the present study, the following conclusions can be drawn:

1. After teeth were immersed in tea solution, in comparison with the baseline values, both of the evaluated universal shade resin composite materials had unsatisfactory color matching values in class V restorations.
2. Combining instrumental and visual methods to evaluate the shade matching of resin composite materials is useful.
3. Accelerated aging by thermocycling had an obvious negative effect on the microhardness of universal shade resin materials.

## Data Availability

The datasets used and/or analyzed during the current study are available from the corresponding author upon reasonable request.
